# Investigation of Voltage Range and Self‐Discharge in Aqueous Zinc‐Ion Hybrid Supercapacitors

**DOI:** 10.1002/cssc.202002931

**Published:** 2021-02-10

**Authors:** Jie Yang, Mark A. Bissett, Robert A. W. Dryfe

**Affiliations:** ^1^ Department of Chemistry University of Manchester M13 9PL Manchester UK; ^2^ National Graphene Institute University of Manchester M13 9PL Manchester UK; ^3^ Department of Materials University of Manchester M13 9PL Manchester UK

**Keywords:** aqueous zinc ion hybrid supercapacitors, voltage range, self-discharge, high performance

## Abstract

Aqueous zinc‐ion hybrid supercapacitors are a promising energy storage technology, owing to their high safety, low cost, and long‐term stability. At present, however, there is a lack of understanding of the potential window and self‐discharge of this aqueous energy storage technology. This study concerns a systematic investigation of the potential window of this device by cyclic voltammetry and galvanostatic charge–discharge. Hybrid supercapacitors based on commercial activated carbon (AC) demonstrate a wide and stable potential window (0.2 V to 1.8 V), high specific capacitances (308 F g^−1^ at 0.5 A g^−1^ and 110 F g^−1^ at 30 A g^−1^), good cycling stability (10000 cycles with 95.1 % capacitance retention), and a high energy density (104.8 Wh kg^−1^ at 383.5 W kg^−1^), based on the active materials. The mechanism involves simultaneous adsorption–desorption of ions on the AC cathode and zinc ion plating/stripping on the Zn anode. This work leads to better understanding of such devices and will aid future development of practical high‐performance aqueous zinc‐ion hybrid supercapacitors based on commercial carbon materials, thus accelerating the deployment of these hybrid supercapacitors and filling the gap between supercapacitors and batteries.

## Graphical abstract

**A window into self‐discharge**: A systematic investigation of the potential window and self‐discharge in aqueous zinc‐ion hybrid supercapacitors based on commercial activated carbon (AC) and Zn foil with nontoxic electrolyte is carried out by cyclic voltammetry and galvanostatic charge–discharge methods, with a view to filling the gap between supercapacitors and batteries.

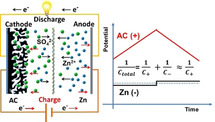

## Introduction

The use of clean renewable energy sources such as solar and wind are of prime importance to the sustainable development of modern society with respect to pressing environmental issues related to our energy use.^[1]^ To achieve this goal, effective and efficient energy storage devices such as batteries and supercapacitors are required. The general properties of different storage devices are determined by the corresponding mechanism of energy storage.^[2]^ Batteries are able to store large amounts of energy but deliver it slowly, resulting in their high energy densities and low power densities.^[3]^ Unlike batteries, supercapacitors (especially electric double‐layer capacitors, EDLCs) store charges at the surface, or near surface, of the active materials and can deliver energy in a short time, thus providing high power densities and low energy densities.^[4]^ Additionally, supercapacitors have much better cycling stability than batteries. Supercapacitors as energy storage devices are very promising in various fields, such as hybrid electric vehicles, smart grids and aircraft, in which high power densities and long lifetimes are needed.^[5]^ However, their wider application has been limited by their relatively low energy densities (<10 Wh kg^−1^).^[6]^ Therefore, various strategies have been adopted to enhance the energy densities of supercapacitors without sacrificing the merits in terms of power densities and cyclability.

The energy density (*E*) of a supercapacitor is determined by the specific capacitance of active materials (*C*) and the square of operating voltage (*U*) based on the energy equation ($E=\frac{1}{2}\;C\;U^{2})$ . To increase the specific capacitance, many different strategies have been used to develop nanostructured carbon materials with high surface area and hierarchical pores, since the specific capacitance of carbon materials is highly dependent on these properties.^[7]^ Additionally, another common way to improve the capacitance is to make composites by combining carbon materials with metal oxides or conductive polymers.^[8]^ Compared with the methods to improve the electrode capacitance, widening the cell voltage is a more efficient means in terms of the improvement of energy density. Essentially, the operating voltage is determined by the stability of the electrolytes. Although a high ionic conductivity can be obtained in acidic and basic aqueous electrolytes, they are highly corrosive and the voltage is usually restricted to 1.0 V due to decomposition of water.^[9]^ Organic electrolytes have been widely used in commercial supercapacitors, because they possess a wider voltage window of 2.5–2.7 V. Environmental and safety issues, along with cost, are major concerns for organic electrolytes.^[10]^ Although the operating window can be further widened to more than 3 V in ionic liquids, and a much higher energy density can be achieved, the device loses the high power due to the high viscosity and associated low ionic conductivity of ionic liquids.^[9]^ Neutral aqueous electrolytes such as group 1 salts of sulfate and nitrate have been considered as promising electrolytes over the past few years due to the wider operating voltage, up to 1.6 V–2.0 V, and less corrosive nature, thus reducing the environmental impact and providing a higher energy density.[^9^, ^11^]

Constructing better energy storage devices not only relies on the properties of the electrode materials and electrolytes but also depends significantly on the configuration of the devices.[^1b^, ^12^] Therefore, much effort has been devoted to combining the advantages of battery‐type electrodes with capacitor‐type electrodes to obtain high energy and power densities as well as good cyclability.^[13]^ Recently, a novel hybrid supercapacitor with a zinc metal anode has been reported by several groups.^[14]^ For example, Dong et al.^[14a]^ reported a novel energy storage system of zinc‐ion hybrid supercapacitors in which activated carbon materials, Zn metal and ZnSO_4_ aqueous solution serve as cathode, anode and electrolyte, respectively. A very high energy density of 84 Wh kg^−1^ and excellent cycling stability were achieved. Wu et al.^[14c]^ used porous carbon derived from chemically activated graphene as the cathode with 3 M Zn(CF_3_SO_3_)_2_ electrolyte in zinc‐ion hybrid supercapacitors, demonstrating an energy density of 106.3 Wh kg^−1^ and a power density of 31.4 kW kg^−1^. Although there have been great advances in achieving high‐performance zinc‐ion hybrid supercapacitor devices by developing various carbon‐based materials, to the best of our knowledge, no detailed study to optimise the potential window has been performed on aqueous hybrid supercapacitors, nor has the effect of self‐discharge processes been considered. Considering that the potential window is critical to the energy output and lifespan of supercapacitors, and high self‐discharge rates compromise their practical value, it is necessary to perform a comprehensive and systematic investigation of the potential window and self‐discharge in the zinc‐ion hybrid supercapacitors.

In this work, we systematically study the voltage range and self‐discharge in aqueous zinc‐ion hybrid supercapacitors comprised of activated carbon cathode, Zn foil anode and ZnSO_4_ aqueous electrolyte solution. The corresponding mechanism and advantages of the hybrid supercapacitor have also been discussed. Furthermore, the self‐discharge is found to be dependent on the initial voltage and it can be significantly suppressed to enhance the charge storage efficiency in this hybrid device compared with symmetric supercapacitors. The hybrid supercapacitor exhibits a wide operating voltage window from 0.2 V to 1.8 V with high specific capacitance (308 F g^−1^ at 0.5 A g^−1^ and 110 F g^−1^ at 30 A g^−1^) as well as good stability with 95.1 % capacitance retention over 10000 cycles. A much higher energy density of about 104.8 Wh kg^−1^ is achieved at the power density of 383.5 W kg^−1^, based on the mass of the active materials. The combination of cost‐effective and nontoxic electrode materials (commercially available zinc metal and activated carbon) with nonflammable aqueous electrolyte is expected to open a new avenue for next‐generation energy storage devices.

## Results and Discussion

**Figure 1:**
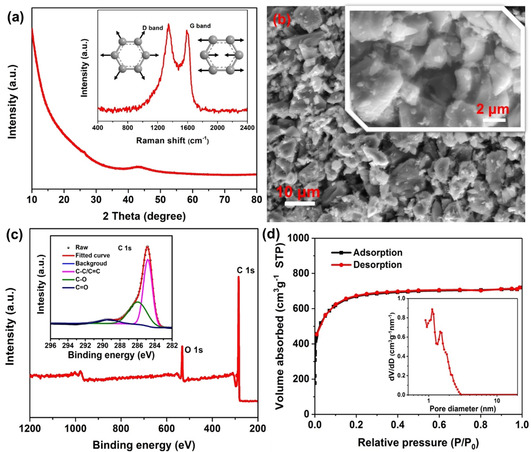
Characterization of YEC‐8 A: a) XRD pattern and Raman spectrum (inset); b) SEM images; c) XPS spectra and high resolution C 1s XPS spectrum (inset); d) nitrogen physisorption isotherms and pore size distribution (inset) derived from NLDFT calculations.

The structure of the activated carbon (AC) denoted as YEC‐8A was characterized by X‐ray diffraction (XRD; Figure 1a). The XRD pattern only shows a relatively broad peak located at 43.1°, which can be ascribed to the (101) plane reflection.^[7e]^ The relatively broad peaks indicate the highly amorphous characteristic of the sample. The Raman spectrum (Figure 1a, inset) also revealed the highly disordered characteristics of the material. The D band at 1346 cm^−1^ corresponds to the breathing mode vibration at free edges and the G band at 1589 cm^−1^ is ascribed to the in‐plane stretching vibration.[^7e^, ^15^] The morphology of YEC‐8A was characterized by scanning electron microscopy (SEM; Figure 1b). The shape of the particles is irregular with relatively coarse surfaces and the particle size is in the range from 5 μm to 20 μm. The surface composition was further identified by X‐ray photoelectron spectroscopy (XPS; Figure. 1c). The elemental composition of the sample surface is comprised of C (ca. 90.17 at. %) and O (ca. 9.83 at. %). The high‐resolution C1s spectrum (Figure 1c, inset) further confirms the presence of surface functional groups such as C−O (ca. 286.2 eV) and C=O (ca. 288.7 eV).^[16]^ The presence of abundant oxygen‐containing groups at the surface of carbon should give rise to hydrophilic properties of the carbon materials, thus facilitating the wettability of electrode and promoting the transport of electrolyte ions.^[17]^

The specific surface area and pore structure of YEC‐8A were probed by nitrogen adsorption‐desorption. The nitrogen adsorption–desorption isotherm of YEC‐8A (Figure 1d) shows the typical characteristics of a type I isotherm.^[18]^ The significant nitrogen adsorption at a relative pressure below 0.01 represents typical feature of micropores (<2 nm), indicating abundant micropores exist in the material. The slight increase in nitrogen adsorption at the relative pressure between 0.05 and 0.3 suggests the lack of abundant mesopores (2 nm–50 nm) in the sample. The specific surface area was calculated to be about 2390 m^2^ g^−1^ based on the Brunauer–Emmett–Teller (BET) model.^[19]^ Such a high specific surface area should provide effective surface sites for ion adsorption and desorption. The pore size distribution was derived from nonlocal density functional theory (NLDFT) based on a slit‐pore model.^[20]^ The corresponding pore size distribution is in the range from 0.87 to 3.03 nm (Figure 1d, inset). The summary is that the pores inside the material consist of many micropores and a small amount of mesopores. Generally, it is believed that both the micropores and mesopores are responsible for charge accumulation and ion adsorption. Furthermore, mesopores can act as channels to transport ions from bulk electrolyte to the electrode/electrolyte interface, facilitating the fast mass transport.^[21]^ In order to effectively and efficiently utilize the surface of the carbon, the relation between the pore size of the carbon electrode and the electrolyte ions should also be considered.^[22]^

**Figure 2:**
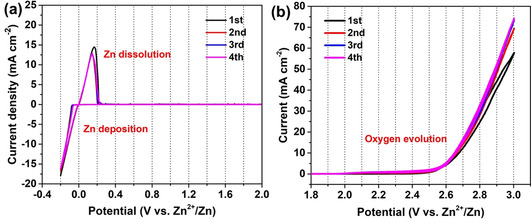
Electrochemical potential window in 2 M ZnSO_4_ electrolyte investigated by cyclic voltammetry at a scan rate of 0.5 mV s^−1^: a) Deposition/dissolution of Zn^2+^; b) oxygen evolution reaction.

The performance of supercapacitors is not only affected by the electrode materials, but also is dependent on the electrolytes used. The electrochemical stability of the electrolytes is critical in determining the operating voltage and lifespan of the supercapacitors. In aqueous supercapacitors with Zn foil electrodes, strong alkaline electrolytes such as KOH solution are not suitable due to the formation of ZnO which is detrimental to the cyclability of the cell. Meanwhile, strongly acidic electrolytes, such as H_2_SO_4_, can react with the Zn metal to generate H_2_. Considering the strongly corrosive and limited electrochemical potential window of alkaline and acidic electrolyte, a mild (neutral) zinc salt is chosen as the electrolyte. To optimize the electrolyte, ZnSO_4_ solutions with different concentrations were prepared and the corresponding ionic conductivities were measured (see the Supporting Information, Figure S1). As the concentration of ZnSO_4_ increases from 1 M to 2 M, the ionic conductivity increases from 49.1 mS cm^−1^ to 58.7 mS cm^−1^. When the concentration is further increased to 3 M, it begins to decrease to 46.5 mS cm^−1^ due to the high viscosity and extensive ion pairing outweighing the effect of additional charge carriers, so 2 M ZnSO_4_ is used as the electrolyte for further experiments. The potential window of 2 M ZnSO_4_ was investigated in a three‐electrode system with stainless steel as the working electrode and Zn foil as both the reference electrode and the counter electrode. As shown in Figure 2a, cyclic voltammetry (CV) indicates that the deposition/dissolution of Zn^2+^ is reversible in ZnSO_4_ solutions. From the CV response, we can roughly determine a stable electrochemical window from 0.2 V to 2.4 V (vs. Zn^2+/^Zn). The oxygen evolution reaction is significantly suppressed up to 2.4 V in this electrolyte (Figure 2b). When the potential is higher than 2.4 V, the parasitic oxygen evolution reaction begins to become quite significant accompanied by the vigorous generation of gas bubbles. The widened potential window can be explained by the strong ion solvation of Zn^2+^ and SO_4_^2−^.^[11d]^ We adopt a conservative estimation of the stable window, which should be stable in the range of 0.2–2.2 V since there are no detectable side reactions in this range.

**Figure 3:**
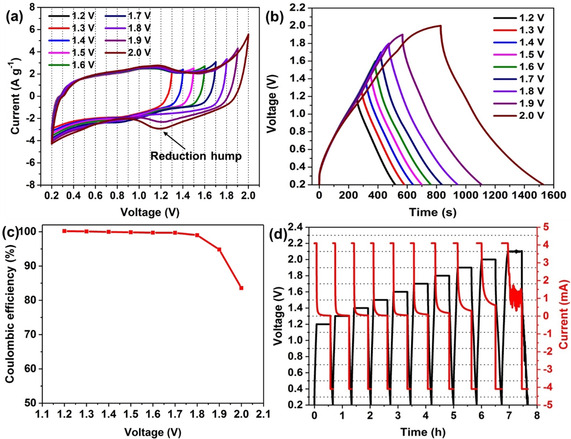
Electrochemical characterizations of AC//ZnSO_4_ (aq)//Zn hybrid supercapacitor: a) Cyclic voltammogram (10 mV s^−1^) of with stepwise increase in positive cell voltage limits; b) GCD characteristics (1 A g^−1^) at different voltage ranges; c) coulombic efficiencies at different voltage ranges; d) GCD and potentiostatic aging profiles at various voltages.

In practical applications, the device is usually employed in a two‐electrode configuration. The available maximum operating voltage was further studied in a two‐electrode configuration (i. e., coin cell) with YEC‐8A as the cathode, zinc foil as the anode and 2 M ZnSO_4_ as the electrolyte. The operating voltage was first investigated by CV over various voltage ranges (Figure 3a). It is observed that a quasi‐rectangular shape of the CV curve is maintained in the range from 0.2 V to 1.8 V. However, when the upper cut‐off voltage is increased to 1.9 V, the current at higher voltage shows a significant increase due to the oxidation of carbon surface functional groups or oxidation of the carbon bulk.^[23]^ Also a “hump” located at around 1.2 V is observed, which is attributed to the electrochemical reduction of quinone surface functionalities to hydroquinone.[^23b^, ^24^] As the upper cut‐off voltage is further increased to 2.0 V, the hump is more pronounced owing to more quinone being generated at high potentials during the anodic sweep. The carbon materials, especially the nanoporous carbon materials with abundant defects, are able to undergo electrochemical oxidation to generate surface oxides (e. g., carboxyl, carbonyl) and even CO/CO_2_ at high potentials.[^23^, ^25^] The highest operation voltage for carbon‐based electrodes can be restricted by such carbon oxidation processes which can occur before the oxygen evolution reaction.^[25]^ Alhough the supercapacitor can still operate beyond the maximum voltage range, the cycle life and the safety are significantly compromised.

The electrochemical potential window was further studied by galvanostatic charge–discharge (GCD) at a current density of 1 A g^−1^(Figure 3b). When the voltage is below 1.9 V, the charge–discharge curves are highly symmetrical, indicating a typical capacitive behavior. As the voltage is further increased to 2.0 V, the linearity of the charge curve is not retained at the highest voltage due to the oxidation of the carbon surface groups. These results are in a good agreement with the CV results. Additionally, the corresponding coulombic efficiencies at different voltages were also calculated (Figure 3c). The coulombic efficiency is defined as the ratio of the discharge capacity to the charge capacity and it is noted that the coulombic efficiency decreases as the voltage increases. The coulombic efficiency is nearly 100 % in the voltage range from 1.2 V to 1.8 V, indicating excellent charge and discharge reversibility of the supercapacitors. In contrast, when the voltage exceeds 1.9 V, the coulombic efficiency drops to 83.6 %. A coulombic efficiency significantly lower than 100 % indicates that additional irrevisible reactions occur at the high voltage range in the charging process. Generally, the practical coulombic efficiency is expected to approach 100 %. Otherwise, both the lifespan and the safety would be undermined because of the irreversible side‐reactions: the operational voltage window should therefore be restricted from 0.2 V to 1.8 V.

The CV and GCD tests can reveal the point at which a side reaction has started to take place. However, if the reaction is thermodynamically feasible, but kinetically controlled, it is difficult to discern the reaction immediately. For this reason, the electrochemical potential window was further confirmed by a combination of GCD and potentiostatic aging. The cell was charged to a set voltage and kept at the corresponding voltage for 30 min to observe the current response and then discharged (Figure 3d). The final dwell current response during the potentiostatic dwell was employed to evaluate the electrode stability. If the final dwell current is negligible, it indicates an ideal electric double layer behavior and no Faradaic current. When the cell is kept in the voltage range from 1.2 V to 1.8 V, the final current during the dwell period tends to zero. As the voltage increases to 2.0 V, the response current is much higher than zero, suggesting a slow pseudocapacitive current under this voltage. As the voltage is further increased to 2.1 V, the strong oscillation of the response current indicates a drastic Faradaic reaction and the failure of the cell under this high voltage.

**Figure 4:**
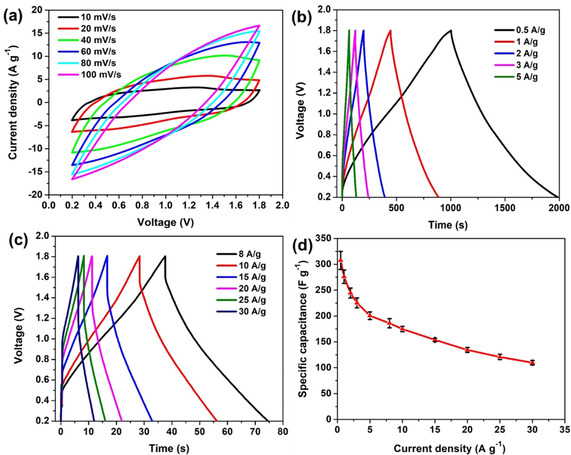
a) Cyclic voltammograms as a function of scan rate; b, c) GCD curves at various current densities; d) specific capacitances at various current densities.

After confirmation that the stable operating voltage range of the cell is from 0.2 V to 1.8 V, the electrochemical behavior was further investigated by CV at various scan rates (Figure 4a). The CVs show no distinct redox peaks, suggesting it displays the approximate characteristics of a classical double layer capacitor. The CVs do not coincide at different scan rates owing to the diffusion limitation of the ions. The corresponding GCD curves are shown in Figure 4b and c. The linearity of the responses obtained at various current densities demonstrates that the device shows typical capacitive behavior. The increase in IR drop with current density also has a detrimental influence on the electrochemical performance. The specific capacitances at different current densities were calculated based on the discharge process. As shown in Figure 4d, a high specific capacitance, of about 308 F g^−1^, is obtained at a low current density of 0.5 A g^−1^. As the current density increases to 1, 2, 3, and 5 A g^−1^, the capacitance decreases to 276, 244, 225 and 201 F g^−1^, respectively. At higher current densities of 8, 10, 15, 20, 25 and 30 A g^−1^, the corresponding specific capacitances are 186, 175, 154, 134, 121, and 110 F g^−1^, respectively. The low capacitance retention of 35.69 % at 30 A g^−1^ should be ascribed to the slow ionic diffusion in the ZnSO_4_ solution as well as relatively slow stripping and plating process on the Zn electrode.

**Figure 5:**
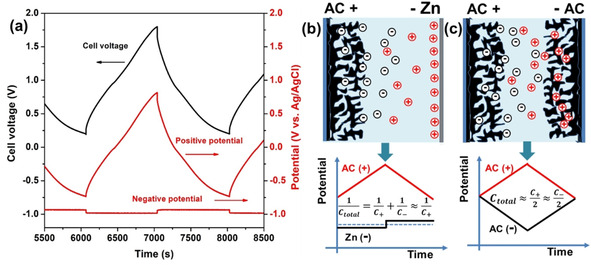
a) GCD profiles recorded at 0.5 A g^−1^ in a two‐electrode system with a Ag/AgCl reference electrode. b,c) Schematic diagram for AC−Zn hybrid supercapacitors (b) and AC‐AC symmetric supercapacitors (c).

In order to understand the mechanism of the hybrid supercapacitors, the potential profiles of both the AC electrode and Zn electrode were simultaneously recorded by using a Ag/AgCl reference electrode in an open two‐electrode system. The zinc‐ion hybrid supercapacitor was characterized using GCD at 0.5 A g^−1^ (Figure 5a). The potential profile of the AC cathode exhibited an approximately linear behavor with the charge time, indicating a capacitive behavior. The zinc metal anode shows a constant potential of −0.983 V (vs. Ag/AgCl) for deposition of Zn^2+^ and −0.933 V (vs. Ag/AgCl) for stripping of Zn. The behavior of the zinc metal electrode is similar to that of a battery electrode. Considering that the potential range of the zinc electrode is almost unchanged, its specific capacitance can be considered as semi‐infinite compared with the specific capacitance of the AC electrode. As this hybrid capacitor consists of an AC electrode and zinc electrode connected in series, the total capacitance is dominated by the capacitance of the smaller (i. e., AC) electrode.^[26]^ So the total capacitance of this device is approximately equal to that of the AC electrode: if the weight and thickness of zinc anode is optimised, this leads to the effective utilization of the capacitance of the AC electrode in this hybrid supercapacitor. By constrast, in the case of a symmetric supercapacitor, the specific capacitance of a cell is restricted to approximately 25 % of that of a single electrode. Since the AC electrodes consist of the activated carbon, conductive carbon, binder and current collector, we can directly use zinc metal to replace one of the AC electrodes in a symmetric supercapacitor to construct a hybrid supercapacitor in which the zinc metal can serve as the active material and current collector without additional binders and conductive agent. Therefore, this strategy may provide a possibility to fully utilize the capacitance of AC electrode at the cell level and potentially reduce the total weight of the cell.

To further confirm that the specific capacitance is dominated by AC electrode, we have chosen another kind of activated carbon known as C9157 with a low specific surface area of 915 m^2^ g^−1^ as the cathode (Figure S4). It delivers a relatively low specific capacitance of 100 F g^−1^ at a current density of 0.5 A g^−1^ (Figure S6), which testifies that the performance of the cell is dominated by the AC electrode and the corresponding specific capacitance is highly dependent on the specific surface area of the activated carbon. The physical and chemical nature of carbon materials clearly has a great influence on the adsorption–desorption of ions at the interface between the carbon and electrolyte. Therefore, the rational design of nanostructured carbon materials is expected to optimize the performance of the hybrid supercapacitors.

**Figure 6:**
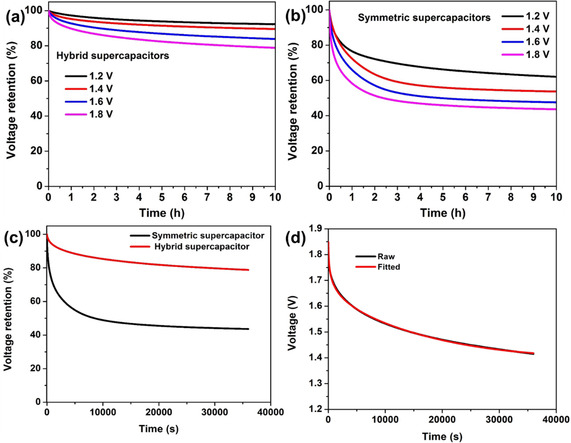
a, b) Voltage retention of zinc‐ion hybrid supercapacitors (a) and symmetric supercapacitors (b) at different initial voltages under open‐circuit conditions. c) Comparison of the voltage retention at an initial voltage of 1.8 V between zinc‐ion hybrid supercapacitors and symmetric supercapacitors. d) Fitting of the self‐discharge of zinc‐ion hybrid supercapacitors based on a mixed mechanism.

Although considerable efforts have been made to improve the energy and power densities of supercapacitors, the self‐discharge of hybrid supercapacitor has been comparatively overlooked. Self‐discharge means the spontaneous voltage drop of energy storage devices over a period of storage time, thus resulting in energy loss of the devices.^[27]^ In batteries, the self‐discharge rate is relatively slow due to the energy being stored through the bulk of the electrode materials. In electrical double‐layer capacitors, the mechanism is usually based on the electrostatic adsorption of ions on the interface between the electrode and electrolyte, leading to a much faster self‐discharge rate. Considering that self‐discharge is a significant concern in the practical application of supercapacitors, the self‐discharge phenomenon of this hybrid supercapacitor were systematically investigated. The cell was charged to a given volage with a low current density of 0.5 A g^−1^ and then held for 20 min to keep it fully charged. The self‐discharge voltage profiles for various initial voltages are shown in Figure 6a. When the zinc‐ion hybrid supercapacitors are charged to 1.2, 1.4, 1.6, and 1.8 V, the retention of voltage is 92.29 %, 89.53 %, 83.86 %, and 78.79 %, respectively, after 10 h under open circuit conditions. For comparison (Figure 6b), the symmetric supercapacitors were also charged to 1.2, 1.4, 1.6, and 1.8 V, with corresponding voltage retentions of 62.04 %, 53.70 %, 47.53 %, and 43.64 %, respectively. There is a distinct difference in the voltage retention between the zinc‐ion hybrid supercapacitor and the symmetric supercapacitor (Figure 6c), indicating effective suppression of self‐discharge in this hybrid supercapacitor due to the higher energy barrier for spontaneous stripping/plating of zinc in comparison with the electrostatic adsorption–desorption of ions. The self discharge is driven by the system minimsing its Gibbs energy and is sensitive to the initial voltage.^[28]^ Considering that supercapacitors will lose 75 % of the stored energy once their voltage drops to half of the initial voltage, the rapid loss of voltage can cause fatal damage to the performance of supercapacitors.^[27b]^ These results prove that the self‐discharge phenomena were substantially suppressed in the hybrid supercapacitors. The merit of this hybrid configuration in suppressing self‐discharge has also been demonstrated by Zhi et al.^[29]^ For instance, the voltage retention in the phosphorene‐Zn hybrid capacitor was 72.73 % within 15 h under the initial voltage of 2.2 V and the voltage retention was 65.93 % after 72 h in the Ti_3_C_2_‐Zn hybrid capacitor fully charged to 1.35 V. Since self‐discharge is an inevitable issue in supercapacitors, various methods have been used to suppress it by tailoring the electrode,^[30]^ introducing additives to the electrolyte,[^28b^, ^31^] and using ion‐exchange membranes as separators.^[31a]^ Compared with reported methods, this hybrid configuration is a simpler and more effective strategy, without side effects, to alleviate the self‐discharge phenomena in supercapacitors. Generally, three different self‐discharge mechanisms have been proposed based on electric double‐layer capacitors. The first is ohmic leakage, which takes place through internal resistance present in the device.[^27b^, ^28a^] The second is charge redistribution through diffusion of ions adsorbed at the electrode surface.^[32]^ The third is a faradaic process, owing to oxidation or reduction of redox species and impurities on the electrode surface.[^27b^, ^33^] As to ohmic leakage, the relationship between voltage and time is similar to that for a dielectric capacitor [Equations (1) and 2]:[^28a^, ^34^]

(1) $$U_{t}=U_{0}exp\left(-\frac{t}{RC}\right)$$

(2) $$\mathrm{or}\;\ln U_{t}=\ln U_{0}-\frac{t}{RC}$$

where *U_t_* is the device voltage during the self‐discharge process, *U*_0_ is the initial voltage, *t* is the self‐discharge time, *C* is the capacitance of the cell, and *R* is internal resistance in the cell. For self discharge under diffusion control, the voltage variation is assumed to be a function of the square root of time [Equation 3]:[^27a^, ^28^]

(3) $$U_{t}=U_{0}-m\sqrt{t}$$

where *m* is a constant related to the diffusion parameter of the ions near the electrode surface. For self‐discharge caused by faradaic reactions, which may be attributed to local overcharging or impurities, the voltage variation can be expressed by Equation 4:[^27a^, ^28a^, ^32a^, ^34^]

(4) $$U_{t}=U_{0}-\frac{RT}{\alpha F}\ln\frac{\alpha Fi}{RTC}-\frac{RT}{\alpha F}\ln\left(t+\frac{CK}{i}\right)$$

where *R* is ideal gas constant, *T* is temperature, α represents charge transfer coefficient, *F* is the Faraday constant, *i* is the exchange current, and *K* represents an integration constant. In conventional dielectric capacitors, the self‐discharge is dominated by the leakage mechanism, but voltage changes may be caused by hybrid mechanisms in electrochemical capacitors. Combining the three possible mechanisms, the general relation of voltage and time can be described by Equation 5:[^27a^, ^28b^]

(5) $$U_{t}=U_{0}\exp\left(-\frac{t}{RC}\right)-m\sqrt{t}-a\ln\left(t+\frac{CK}{i}\right)+b$$

where *a*, and *b* are constants.

When the ohmic leakage model is used to fit the self‐discharge curve, it is found that the self‐discharge phenomenon cannot be entirely attributed to ohmic leakage, since the self discharge does not exhibit a purely exponential decrease (Figure S7). Addtionally, the diffusion process and faradaic process are also considered to explain the self‐discharge process. It is difficult to match a single model with the practical self‐discharge behavior (Figures S8 and S9). When the three mechanisms are combined, it is possible to obtain a good match between the test curve and fitting results in the whole period (Figure 6d). This implies that the self discharge can be ascribed to the joint effect of the three mechanisms.

**Figure 7:**
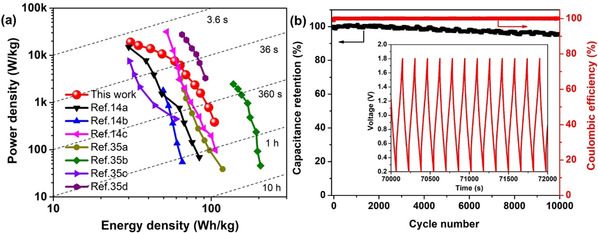
(a) Ragone plots of the Zn‐ion hybrid supercapacitor based on the mass of active materials compared to the reported results. (b) Cycling stability at 4 A g^−1^ for 10000 cycles.

The Ragone plot based on the mass of active materials is shown in Figure 7a. A high energy density of 104.8 Wh kg^−1^ was achieved at the power density of 383.5 W kg^−1^ and a high power density of 19.0 kW kg^−1^ was obtained at the energy density of 30.8 Wh kg^−1^, which are superior to those of most reported zinc‐based hybird supercapacitors.[^14^, ^35^] This level of performance confirms the effectiveness of the hybrid configuration design to enhance energy storage. In view of the total weight of the device including active materials, binder, current collectors, electrolyte, separator and packaging, the Ragone plots merely based on the mass of active materials could not realistically present the practical performance in energy and power. Generally, the proportion of the active materials is about 30 % of the total mass in a packaged commercial supercapacitor. So a factor of 3–4 is usually employed to reappraise the practical performance of the active materials in energy and power.^[3]^ Therefore, the energy densities of the assumed packaged supercapacitors are expected to be about 25 to 30 Wh kg^−1^, which are higher than those of commercial activated carbon based supercapacitors (5 to 10 Wh kg^−1^). Additionally, the cycling stability of the cell was evaluated by using the GCD method at 4 A g^−1^ (Figure 7b). This hybrid supercapacitor has exhibited excellent long‐term stability with a capacitance retention of about 95.1 % over 10000 cycles with almost 100 % coulombic efficiency. Considering that the aqueous mild electrolyte is nonflammable, noncorrosive and eco‐friendly, aqueous zinc‐ion hybrid supercapacitors are expected to hold great potential in future practical applications to fill the gap between electrochemical capacitors and batteries.

## Conclusion

In summary, the voltage range and self‐discharge phenomena have been systematically investigated in aqueous zinc‐ion hybrid supercapacitors, which leads to a better understanding of this hybrid device. Three universal approches based on CV and GCD techniques were employed to thoroughly investigate the voltage window in this aqueous zinc‐ion hybrid supercapacitor based on 2 M ZnSO_4_. The corresponding mechanism and configuration advantages of this hybrid supercapacitor have also been discussed. The mechanism of the zinc‐ion hybrid supercapacitor involves the simultaneous adsorption–desorption of ions on the AC cathode and zinc ion plating/stripping on the Zn anode. Constructed by the large capacity of the Zn metal negative electrode, with its relatively low standard redox potential of −0.76 V (vs. standard hydrogen electrode) and neutral aqueous electrolyte, the hybrid supercapacitors have demonstrated excellent electrochemical performances including high specific capacitance (308 F g^−1^ at 0.5 A g^−1^ and 110 F g^−1^ at 30 A g^−1^), good cycling stability (10000 cycles with 95.1 % capacitance retention) and a high energy density 104.8 Wh kg^−1^ at 383.5 W kg^−1^ (30.8 Wh kg^−1^ at 19.0 kW kg^−1^) based on the active materials, which exceed those of most reported zinc‐based hybrid supercapacitors and symmetrical supercapacitors. Additionally, self discharge was substantially suppressed in the hybrid supercapacitors compared with the symmetric supercapacitors, a phenomenon which is highly dependent on the initial voltage. Since the zinc foil can be simultaneously used as both current collector and active material, the unnecessary weight and volume of the devices can potentially be reduced to some extent. The performance of this device can be further boosted by developing novel advanced carbon‐based materials or other composites. This work is expected to provide more insight into the hybrid supercapacitors and accelerate industrial development of high‐voltage aqueous hybrid supercapacitors for next‐generation energy storage devices.

## Experimental Section

### Materials and chemicals

The activated carbon (AC) (YEC‐8 A) was purchased from Fuzhou Yihuan Carbon Co.,Ltd (China). Zinc foil (thickness of 180 μm), ZnSO_4_ ⋅ 7H_2_O, conductive carbon black (Super P with sizes from 40 to 100 nm) and cellulose separator were purchased from Alfa Aesar manufacturer. Activated charcoal (C9157) and 60 wt % polytetrafluoroethylene (PTFE) dispersion in H_2_O were purchased from Sigma‐Aldrich Company. After polishing, Zinc foil (15 mm diameter) was directly used as the anode. The cathode was composed of activated carbon, conductive carbon black and PTFE in the mass ratio of 8 : 1 : 1. *Iso*‐propanol was added to the above mixture and the resultant suspension was rolled into thin sheets of about 100 μm thickness. The sheets were then punched into electrodes of 12 mm diameter. All the electrodes were dried in an oven at 80 °C for 24 h. The mass loading of the activated carbon is about 4 mg cm^−2^.

### Materials characterization

The structure of the materials was investigated by powder X‐ray diffraction (Bruker D8 Advanced diffractometer) with Cu_Kα_ radiation (*λ*=1.5406 Å) at 40 kV. The morphology of the materials was observed by using a Philips XL30 field emission scanning electron microscope (FESEM) at 5 kV. Raman spectroscopy was recorded using a Renishaw inVia microscope with an excitation wavelength of 532 nm at a power of approximately 1 mW. X‐ray photoelectron spectroscopy (XPS) was performed on a Kratos Axis Ultra spectrometer with a mono‐chromatic Al_Kα_ X‐ray source. Nitrogen adsorption–desorption measurements were used to characterize the pore structure on a Micromeritics ASAP 2020 analyzer at 77 K. The specific surface area was derived from the Brunauer‐Emmett‐Teller (BET) model and the pore size distribution (PSD) was derived from a nonlocal density functional theory (NLDFT) model.[^19^, ^20^]

### Electrochemical measurements

The cyclic voltammetry (CV) was carried out in a PGSTAT302 N potentiostat (Metrohm Autolab). The galvanostatic charge–discharge (GCD) tests were performed in a Battery Test System (BaSyTec GmbH, Germany). The three‐electrode tests were performed with a stainless steel (Type 316; ca. 2 cm^2^) working electrode, zinc foil as both the reference electrode and as the counter electrode. The stainless‐steel working electrode was employed to make the three electrode experiments consistent with the conditions of the coin cell work, where a stainless‐steel casing can come into contact with the electrolyte. For the hybrid supercapacitors, the cells were assembled into a CR2032 coin‐cell type device with activated carbon electrode as cathode, a zinc anode and 2 M ZnSO_4_ aqueous electrolyte. The cyclic voltammetry was performed at various scan rates ranging from 10 to 100 mV s^−1^. GCD tests were carried out with a voltage range from 0.2 V to 1.8 V at various current densities. Moreover, the cyclic stability was performed at a current density of 4 A g^−1^ for 10000 cycles. All the electrochemical tests were carried out at room temperature.

According to the galvanostatic discharge curve, the gravimetric capacitance (*C*) was calculated by using Equation 6:

(6) $$C=\frac{I\Delta t}{m\Delta V}$$

where *I* is the discharge current, *▵t* represents the discharge time, *m* is the mass of the activated carbon and *▵V* is potential range.

The corresponding energy density (*E*) and power density (*P*) were calculated by using Equations (7) and (8) respectively:

(7) $$E=\int_{0}^{\Delta t}V\left(t\right)Idt$$

(8) $$P=\frac{E}{\Delta t}$$

Where Δ*t* is the discharge time, $V\left(t\right)$ is the voltage and *I* is the current density. The detailed calculation procedure is provided in the Supporting Information.

## Conflict of interest

The authors declare no conflict of interest.

## Supporting information

As a service to our authors and readers, this journal provides supporting information supplied by the authors. Such materials are peer reviewed and may be re‐organized for online delivery, but are not copy‐edited or typeset. Technical support issues arising from supporting information (other than missing files) should be addressed to the authors.

Supplementary
