# Supplementary material for: Investigation of Voltage Range and Self‐Discharge in Aqueous Zinc‐Ion Hybrid Supercapacitors
Source: ChemSusChem. 2021 Feb 10;14(7):1700–9. doi: 10.1002/cssc.202002931 (PMC8048863; doi:10.1002/cssc.202002931)
Supplement: Supplementary file 1 — Supplementary [file CSSC-14-1700-s001.pdf]

ChemSusChem

Supporting Information

Investigation of Voltage Range and Self-Discharge in Aqueous Zinc-Ion Hybrid Supercapacitors

Jie Yang, Mark A. Bissett,* and Robert A. W. Dryfe*© 2021 The Authors. ChemSusChem published by Wiley-VCH GmbH. This is an open access article under the terms of the Creative Commons Attribution License, which permits use, distribution and reproduction in any medium, provided the original work is properly cited.

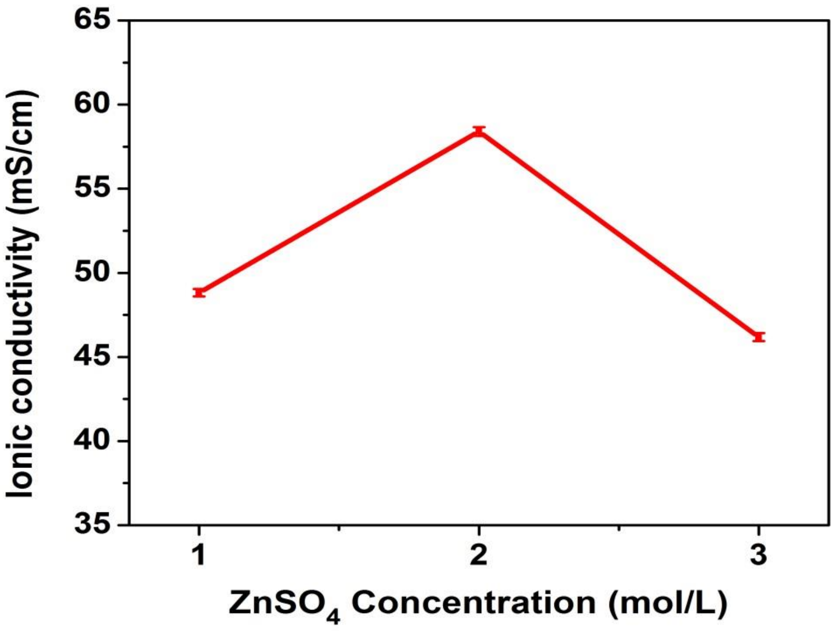

Figure S1. Ionic conductivities of $ZnSO_4$ measured for the concentrations used in this study.

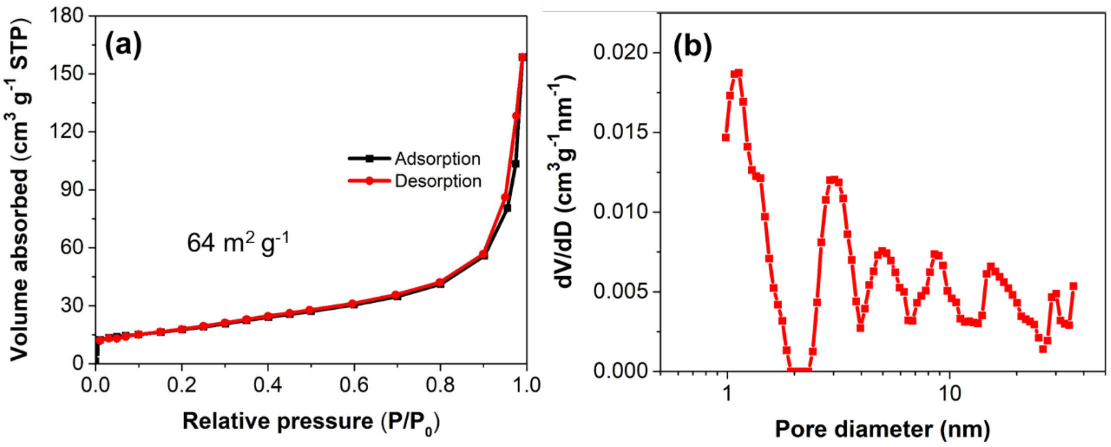

Figure S2. Conductive carbon black (Super P): (a) Nitrogen physisorption isotherms and (b) NLDFT-derived pore size distribution.

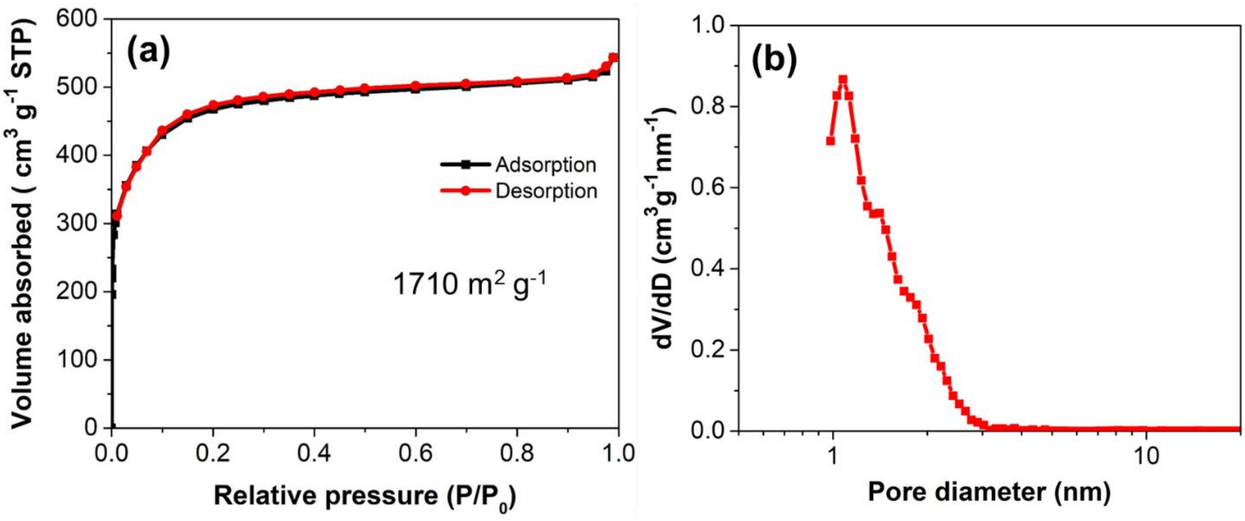

Figure S3. Fabricated electrode using the activated carbon (YEC-8A) as active materials: (a) Nitrogen physisorption isotherms and (b) NLDFT-derived pore size distribution.

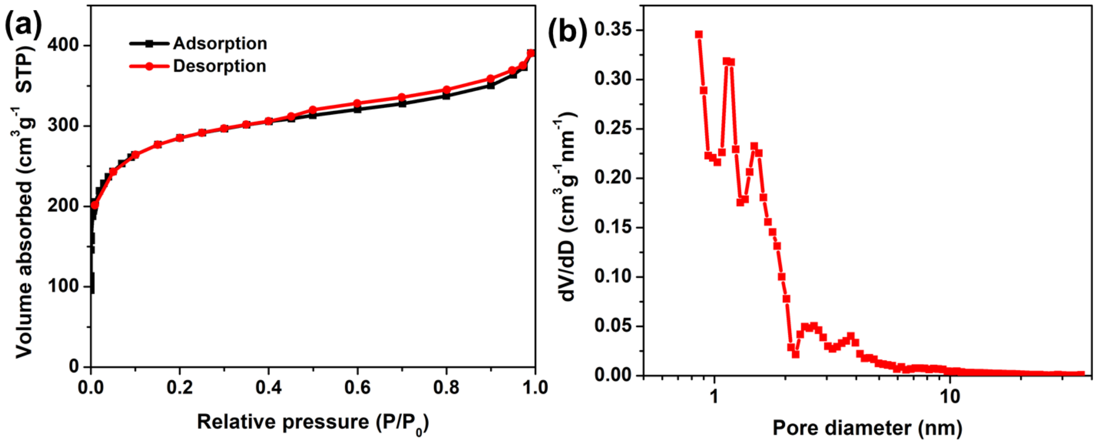

Figure S4. Activated charcoal (C9157): (a) Nitrogen physisorption isotherms and (b) NLDFT-derived pore size distribution.

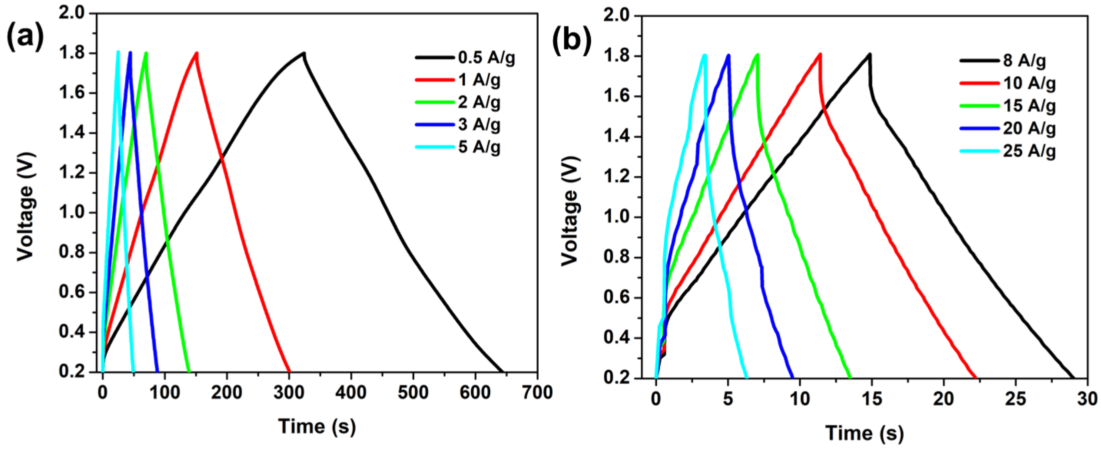

Figure S5. Representative galvanostatic charge-discharge profiles of zinc ion hybrid supercapacitors based on activated charcoal (C9157).

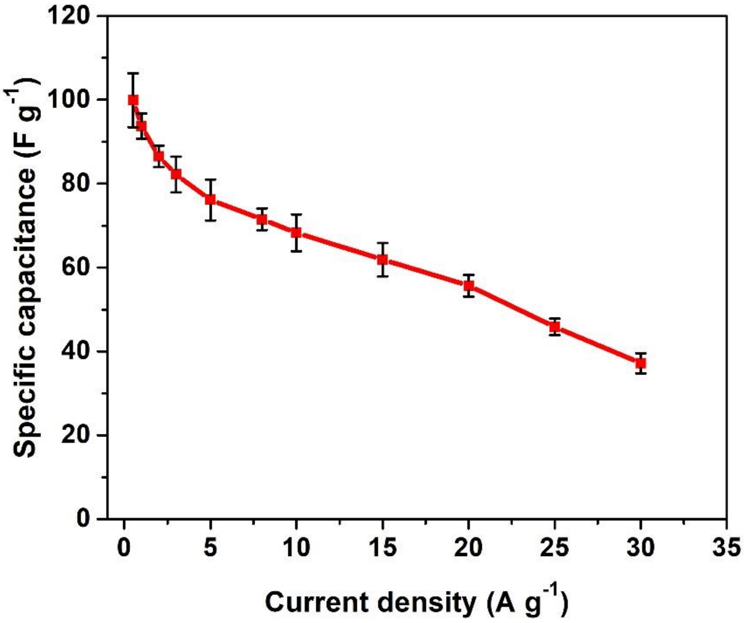

Figure S6. Specific capacitances of activated charcoal (C9157) at various current densities

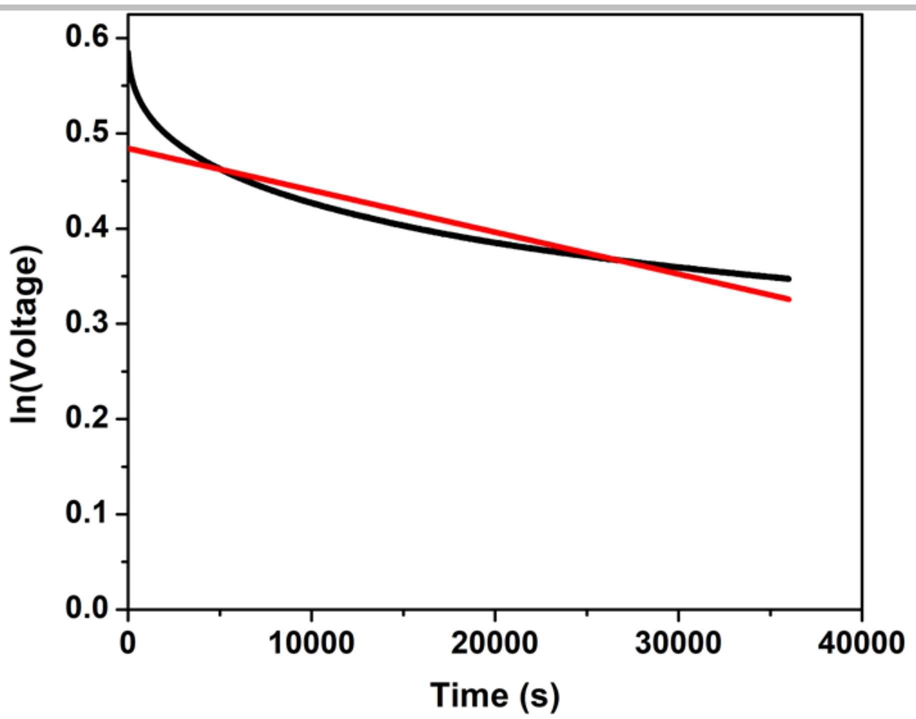

Figure S7. Fitting of self-discharge process based on ohmic leakage model.

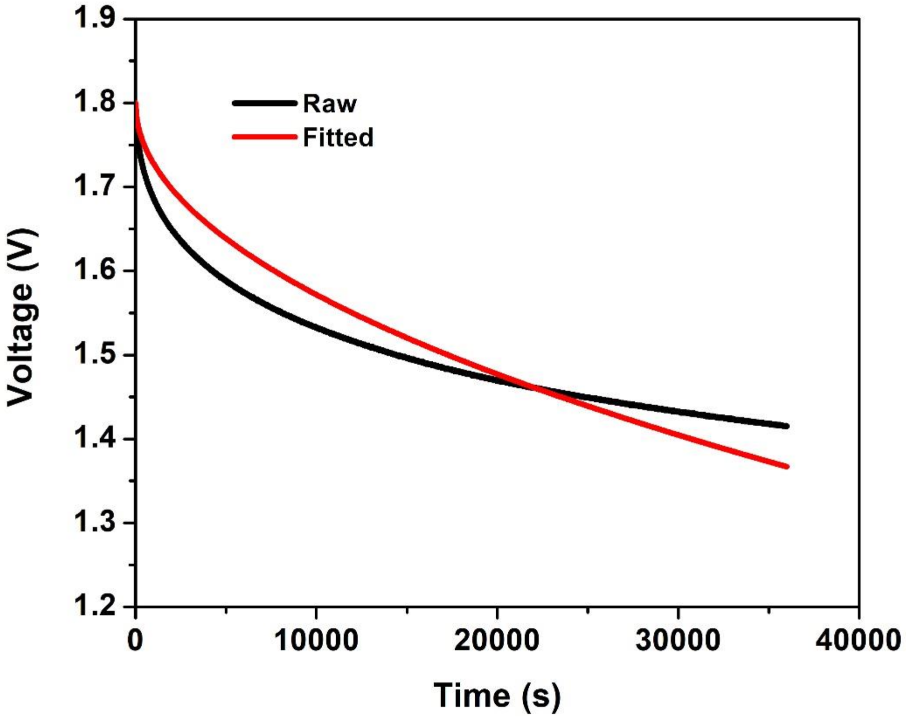

Figure S8. Fitting of self-discharge process based on ion diffusion model.

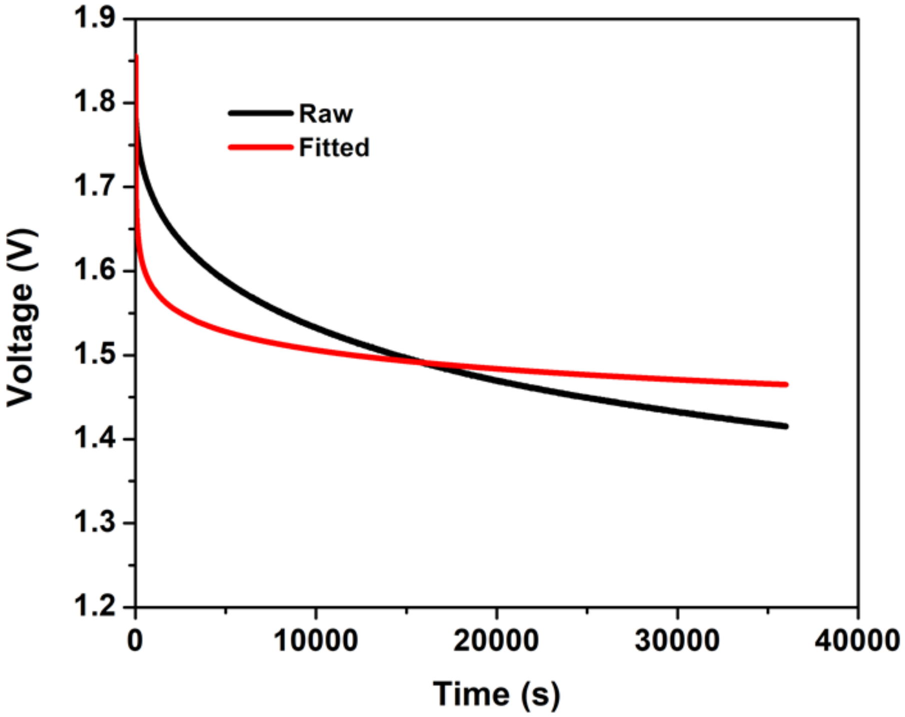

Figure S9. Fitting of self-discharge behavior based on faradaic process.

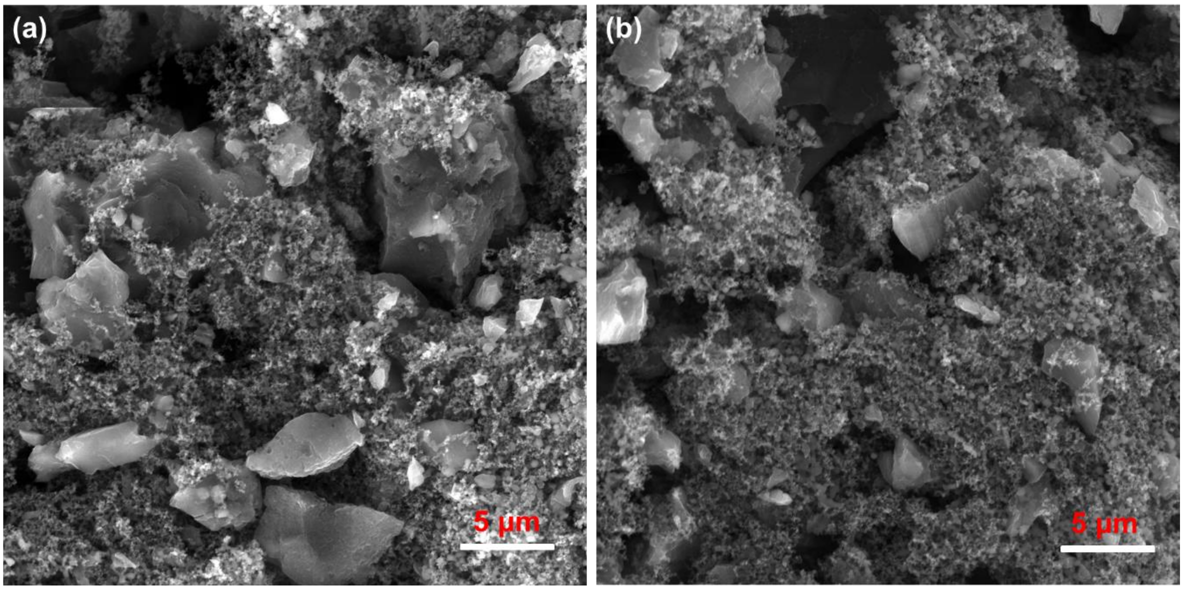

Figure S10. SEM images of the AC electrode (a) before and (b) after cycling.

Potential conversion

The standard redox potential of Zn^{2+}/Zn is as follows:

$$\mathrm{Zn^{2+}} + 2\mathrm{e^-} \rightleftharpoons \mathrm{Zn}\ (E^\circ = -0.762\ \mathrm{V\ vs.\ SHE})$$

The practical concentration of Zn^{2+} is 2 mol L^{-1}. According to the Nernst equation, the practical potential of Zn^{2+}/Zn can be calculated as following:

$$E = E^\circ - \frac{RT}{nF}\ln\frac{[\mathrm{Zn}]}{[\mathrm{Zn^{2+}}]} = -0.762 - \frac{8.314\times298.15}{2\times96485}\times\ln\frac{1}{2} = -0.754\ \mathrm{V\ (vs.\ SHE)}$$

The standard potential of reference electrode (Ag/AgCl with saturated KCl solution) is 0.222 V (vs. SHE). Therefore, the required theoretical potential of (Zn^{2+}/Zn) relative to the reference electrode (Ag/AgCl) is estimated to be -0.976 V (Vs. Ag/AgCl). This value is consistent with the experimentally obtained values.

Calculation of energy density and power density

Generally, the energy density of supercapacitors can be estimated by numerically integrating the discharge curves:[1]

$$E = \int_{t_1}^{t_2} IV dt \qquad (1)$$

Where $t_1$and t_2 are time, I is the current density, V is the discharge voltage.

If V is a liner function of time t, then

$$E = \int_{t_1}^{t_2} IV dt = \frac{1}{2} C({V_2}^2 - {V_1}^2) \qquad (2)$$

where V_1 is minimum voltage at time t_1, V_2 is maximum voltage at time t_2 and C is the capacitance of the device.

If V_1 is set to be 0 V which is the common minimum voltage in symmetric supercapacitors, then equation (2) can be transformed into the most common equation widely used to calculate energy density in supercapacitors,

$$E = \frac{1}{2} C{V_2}^2 = \frac{1}{2} C\Delta V^2$$

Where ΔV is the voltage window $(\Delta V = V_2 - V_1)$.

However, in the case of a hybrid supercapacitor, the minimum voltage V_1 for capacitive behavior is usually larger than 0 V. Therefore, the equation (2) is used to calculate the corresponding energy density. It should be noted that the voltage does not display an ideal linear dependence on time in hybrid supercapacitors. If the equation is directly used to calculate the energy density in a hybrid device, the corresponding energy density will be overestimated. Therefore, direct numerical integration of the discharge curves is recommended. The differences between these two methods are compared below.

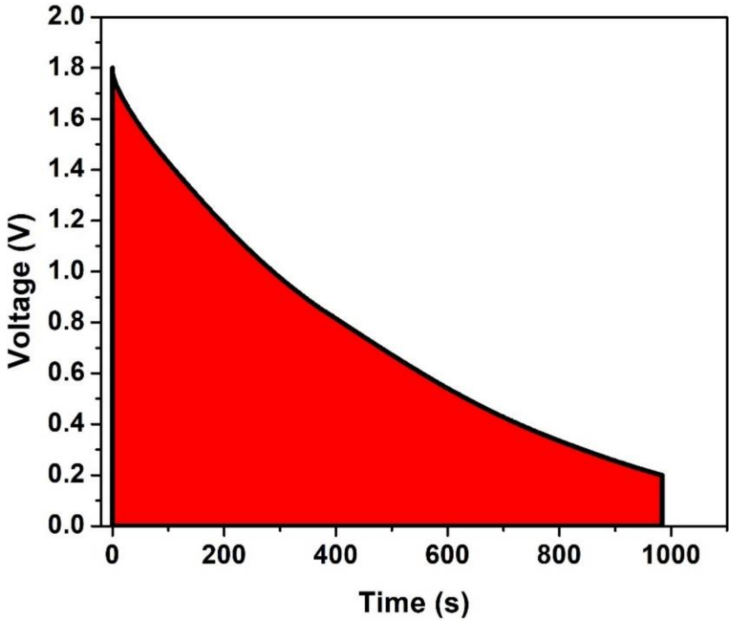

Figure S11. The typical discharge curve of zinc ion hybrid supercapacitor at 0.5 A g^{-1}

The energy density (E) is calculated by using the following equation:

$$E = \int_0^{\Delta t} V(t) I dt = A_S I$$

Where Δt is the discharge time, $V(t)$ is the voltage, I is the current density, A_S is the integral area denoted in the red area.
The energy density (P) is calculated by:

$$P = \frac{E}{\Delta t}$$

Table S1. Comparison of two methods to calculate the energy densities and power densities

Direct integral		Formula method	
$E = \int_{t_1}^{t_2} IV dt$		$E = \frac{1}{2}C({V_2}^2 - {V_1}^2)$	
Energy density (Wh kg^{-1})	Power density (W kg^{-1})	Energy density (Wh kg^{-1})	Power density (W kg^{-1})
104.8	383.5	136.7	500
96.4	785.6	122.7	1000
85.6	1575.2	108.6	2000
78.7	2358.0	100.2	3000
69.0	3869.7	89.1	5000
63.5	6147.9	82.7	8000
58.7	7549.6	77.7	10000

References:

[1] Y. Wang, Y. Song, Y. Xia, *Chem Soc Rev* **2016**, *45*, 5925-5950.
